# Evaluating the implementation of the Saving Babies Lives Care Bundle Version 2 from service user and healthcare professionals’ perspectives: a questionnaire study

**DOI:** 10.1136/bmjoq-2025-003456

**Published:** 2025-09-02

**Authors:** Kate Widdows, Holly Reid, Debbie M Smith, Rebecca Wood-Harper, Elizabeth Camacho, Stephen A Roberts, Alexander E P Heazell

**Affiliations:** 1 Maternal and Fetal Health Research Centre, The University of Manchester Faculty of Biology Medicine and Health, Manchester, UK; 2 Greater Manchester Mental Health NHS Foundation Trust, Manchester, UK; 3 Manchester Academic Health Science Centre, Manchester, UK; 4 Manchester Centre for Health Psychology, The University of Manchester Faculty of Biology Medicine and Health, Manchester, UK; 5 Institute of Population Health, Faculty of Health and Life Sciences, University of Liverpool, Liverpool, UK; 6 Centre for Biostatistics, Institute of Population Health, The University of Manchester Faculty of Biology Medicine and Health, Manchester, UK; 7 Manchester University NHS Foundation Trust, Manchester Academic Health Science Centre, Manchester, UK

**Keywords:** Obstetrics and gynecology, Health services research, Maternal Health Services, Qualitative research, Patient Care Bundles

## Abstract

**Introduction:**

The Saving Babies’ Lives Care Bundle (SBLCB) was introduced in England in 2015 and was updated in 2019 (SBLCBv2). This study aimed to describe the degree to which SBLCBv2 was implemented in practice and describe contemporary experiences of receiving and delivering antenatal and intrapartum care informed by the recommendations of SBLCBv2.

**Methods:**

This cross-sectional questionnaire study was conducted in 28 National Health Service maternity units across England between October and December 2023. The study had two arms, one for maternity service users and one for healthcare professionals. Maternity service users aged ≥16 years who had given birth in the last 12 months were invited to participate in an online survey which contained closed questions about elements of the SBLCBv2, and two free-text questions about their experiences of receiving antenatal and intrapartum care. Maternity healthcare professionals from participating sites were invited to complete a separate questionnaire about delivering care. Responses were summarised by descriptive statistics.

**Results:**

1140 women and 633 healthcare professionals participated. The majority of staff reported implementing all five elements of SBLCBv2, though this varied from 57% (prevention of preterm birth) to 99% (smoking cessation). Service users frequently reported receiving interventions that were part of SBLCBv2: 26% were offered Aspirin and 97% monitored fetal movement. Staff generally reported positive experiences of implementing the SBLCBv2, feeling that it supported clinical decision making. 89% and 86% of service users reported a positive experience in pregnancy and labour, respectively. This was underpinned by positive staff attitudes, behaviours and communication, and being listened to and involved in decisions about care.

**Conclusions:**

SBLCBv2 has been integrated into clinical practice, though some elements require additional focus to increase implementation (e.g., preterm birth). Maternity staff may benefit from additional training to discuss the reasons for and results of interventions to reduce the risk of pregnancy complications.

**WHAT IS ALREADY KNOWN ON THIS TOPIC**

- The Saving Babies’ Lives Care Bundle (SBLCB) is a programme designed to reduce stillbirths; the first iteration was released in 2015, with an update in 2019 which additionally aimed to address preterm births (SBLCBv2). Stillbirth rates in England fell from 2013 to a historic low level in 2020, though progress has since stalled. There have been no studies of the impact of the SBLCBv2 on service users’ experience.

**WHAT THIS STUDY ADDS**

- This study demonstrates that the second iteration of the SBLCB has been integrated into clinical practice with staff and service users reporting recommended interventions being implemented. The majority of service users were satisfied with their antenatal and intrapartum care, though their experience was affected by attitudes and behaviour of staff which may be informed by SBLCBv2 recommendations.

**HOW THIS STUDY MIGHT AFFECT RESEARCH, PRACTICE OR POLICY**

- This research indicates that SBLCBv2 has continued to be implemented into UK maternity care, although some aspects are less well implemented than others and thus require greater focus. Staff training is needed to improve communication about the need for some interventions and ensure service users are actively involved in decision-making.

## Introduction

 Analysis of data from 2000 to 2015 demonstrated that the stillbirth rate in the UK was higher than in comparable nations, and that the annual rate of reduction (ARR) was only 1.4% per year, significantly lower than other nations such as the Netherlands (ARR of 6.8%).^1^ In 2015, the Secretary of State for Health published a national maternity ambition to reduce the number of stillbirths, neonatal deaths, maternal deaths and brain injuries that occur during or soon after birth by 50% by 2025, with an interim reduction of 20% by 2020 to remain on target. To achieve this objective, National Health Service (NHS) England developed the Saving Babies’ Lives Care Bundle (SBLCB), which sought to assist healthcare professionals (HCPs) providing antenatal or intrapartum care to implement recommendations from four areas of established national guidance to address specific risk factors for stillbirth, including: (1) identifying women who smoke cigarettes, using carbon monoxide (CO) screening and support for smoking cessation, (2) improved detection and management of fetal growth restriction (FGR), (3) awareness and management of reduced fetal movements (RFM) and (4) ensuring optimal fetal monitoring in labour to detect intrapartum hypoxia.^2^

Analysis of the SBLCB established that the majority of participating units were able to effectively implement the care bundle.^3^ The evaluation demonstrated a 20% reduction in stillbirth rates 2 years postimplementation of the SBLCB compared with 2 years prior, equating to a 5% ARR. However, this analysis also showed alterations in other important secondary outcomes, including a 6% increase in preterm births prior to 37 weeks’ gestation and a 20% increase in induction of labour (IOL).^3^ There were also important changes in mode of birth, including an increase in both planned and emergency caesarean births. Following the release of the evaluation, a second version of the SBLCB (SBLCBv2) was published in 2019, which added an element on prevention of preterm birth and optimisation of neonatal condition of infants born preterm.^4^ In addition, SBLCBv2 included a section on important principles to be applied when implementing the SBLCBv2 that was developed in collaboration with stakeholder organisations. These included: offering choice and personalised care, promotion of continuity of carer, providing information and implementing national clinical practice guidelines. The components of SBLCBv2 are shown in table 1.

**Table 1:** Overview of SBLCBv2 elements and their associated key interventions

Element	Key interventions
Element 1: Reducing smoking in pregnancy	Carbon monoxide (CO) testing of all women at initial and 36-week antenatal appointment. Referral of those with elevated levels (4 ppm or above) for support from trained stop smoking specialist, based on an opt-out system.
Element 2: Risk assessment, prevention and surveillance of pregnancies at risk of fetal growth restriction (FGR)	Risk assessment and surveillance of women at increased risk of FGR. Assessing women to determine if aspirin should be prescribed using SBLCBv2 algorithm. Risk assessment and management of growth disorders in multiple pregnancy. Surveillance of low-risk populations using symphysis fundal height charts by clinicians trained in their use. When SGA is detected, the frequency of ultrasound review of estimated fetal weight should follow an agreed risk assessment and surveillance pathway. If fetus is <3rd centile initiation of labour and/or delivery should occur between 37+0 and 37+6 weeks. Consider delivery <37+0 weeks if additional concerning features. Fetuses between 3rd–10th centile should be individualised; if no high-risk features, initiation of labour and/or delivery should be offered at 39+0 weeks.
Element 3: Raising awareness of reduced fetal movement (RFM)	Information from practitioners and advice leaflet on RFM to be provided to all pregnant women by 28+0 weeks and RFM discussed at every subsequent contact. Use SBLCBv2 checklist to manage care of women who report RFM.
Element 4: Effective fetal monitoring during labour	All staff who care for women in labour are required to undertake annual training and competency assessment on cardiotocograph (CTG) interpretation and use of intermittent auscultation (IA). Assess risk at the onset of labour to determine the most appropriate fetal monitoring method. At least hourly review of fetal well-being to include: CTG (or IA), reassessment of fetal risk factors, use of a Buddy system to provide objective review for example ‘Fresh Eyes’, a clear guideline for escalation if concerns are raised through the use of a structured process.
Element 5: Reducing preterm births	Assess all women at booking for the risk of preterm birth and stratify to low, intermediate and high-risk pathways. All women are to be offered testing for asymptomatic bacteriuria by sending off a midstream urine for culture and sensitivity at booking. Every provider should have access to transvaginal cervix scanning, a clinician specialing in preterm birth prevention with a clinical pathway for women at risk of preterm birth. Optimise place of birth—women at imminent risk of preterm birth should be offered transfer to a unit with appropriate and available neonatal cot facilities. Antenatal corticosteroids are to be offered to women between 24+0 and 33+6 weeks, optimally at 48 hours before a planned birth. A steroid-to-birth interval of greater than 7 days should be avoided if possible. Magnesium sulfate should be offered to women between 24+0 and 29+6 weeks and considered for women between 30+0 and 33+6 weeks, who are in established labour or are having a planned preterm birth within 24 hours. Ensure neonatal team is involved when a preterm birth is anticipated, so they have time to discuss options with parents before birth and to be present at the birth. For women between 23 and 24 weeks of gestation, a multidisciplinary discussion should be held before birth between the neonatologist, obstetrician and the parents about the decision to resuscitate the baby.
Important principles to be applied when implementing SBLCBv2	Offer choice and personalised care and respect women’s autonomy. Promote the availability of continuity of carer to women. Provide ‘Safe and Healthy Pregnancy Information’ to help women reduce the risks to the baby. Implement best practice in the event of a stillbirth. Inform women of the long-term outcomes of early term birth. Consider how the risks of induction of labour change with gestational age.

SBLCBv2, second version of the Saving Babies’ Lives Care Bundle; SGA, small for gestational age.

In accordance with the quality improvement cycle, NHS England commissioned an evaluation of SBLCBv2. In addition to using routinely collected, nationally reported data, this evaluation used questionnaires and semi-structured interviews (the findings of which are reported elsewhere) to understand service users’ and health professionals’ experiences of implementation of the SBLCBv2. This study aimed to describe the degree to which the SBLCBv2 was implemented in practice and contemporary experiences of receiving and delivering antenatal and intrapartum care.

## Methods

### Participants and procedures

The evaluation was conducted in 28 NHS maternity units across 9 clinical networks in England according to a study protocol.^5^ Maternity units were selected based on their response to an expression of interest to participate in the evaluation, advertised through the Local Maternity Network Systems in March 2023. Sites varied in terms of geography, birth rate and level of neonatal care, ensuring variation in the socioeconomic populations they serve.

Women and birthing people aged 16 years or over who had given birth in England in the last 12 months were invited to participate in an anonymous online survey about their experiences of maternity care and interventions delivered through the SBLCBv2. Women were recruited during their hospital stay prior to discharge via Trust social media and/or from research databases. HCPs who were involved in delivering the SBLCBv2 were invited to participate in an anonymous online survey about their views and experiences of implementing the five care bundle elements. Due to translation constraints, surveys were offered in English only.

The women’s survey contained closed questions about interventions they may have received for each element of the SBLCBv2, and two free-text questions inviting them to comment on their experiences of antenatal and intrapartum care. The HCP survey contained only closed questions about their attitudes and views towards the SBLCBv2, alongside their implementation practices for all five elements of the SBLCBv2. Both surveys are available in online supplemental file 1.

The study was adopted into the National Institute for Health Research Clinical Research Network portfolio and registered on www.clinicaltrials.gov (NCT06453954).

### Patient and public involvement

The protocol was reviewed by our stakeholder advisory group which included representatives from relevant charities and professional organisations. Surveys and participant-facing materials were developed by the research team with consultation from service-users, midwives and HCPs at the Maternal and Fetal Health Research Centre.

### Data analysis

Data from the closed questions are presented descriptively as percentages. Participants were asked to provide explanations for their ratings through two open-ended questions: ‘Why did you feel this way about your antenatal care?’ and ‘Why did you feel this way about your labour and birth?’ Responses to these open questions were analysed independently by three researchers (DMS, KW and RW-H) using an adapted analytical approach. This approach involved first following Krippendorff’s approach to content analysis^6^ and then phases 2–5 of Braun and Clarke’s six-phase process of thematic analysis (2006) whereby codes were reanalysed and presented as themes.^7^

## Results

Completed surveys were obtained from 1140 women and 633 HCPs. The majority of women responded to the open questions, with 1094 responses (96%) for antenatal care and 1067 (94%) for labour and birth. Half of the women accessed the survey through social media (51%). Among the women who responded to the survey (table 2), the majority were white British, UK born (89%), spoke English as their first language (92%) and were more likely to be educated to a degree level or above. Most women had a singleton pregnancy (98%) and gave birth between November 2022 and December 2023, reflecting maternity care received between February 2022 and December 2023.

**Table 2:** Characteristics of the 1140 maternity service users responding to the survey

Characteristic	No. of responses	%
Maternal age (years)		
16–18	5	0.4
19–24	84	7.4
25–29	284	24.9
30–34	450	39.5
35–39	275	24.1
40 or above	42	3.7
Parity		
Primiparous	563	49.4
Multiparous	577	50.6
Ethnicity		
White	985	86.4
Black, black British, Caribbean or African	28	2.5
Asian or Asian British	38	3.3
Mixed or multiple ethnic groups	15	1.3
Other	73	6.4
Mode of birth		
Vaginal birth	490	42.9
Assisted vaginal birth	160	14.0
Emergency caesarean birth	254	22.3
Elective caesarean birth	236	20.7
Induction of labour	446	39.1
Birth outcome		
Alive and well	964	84.6
Admitted to neonatal care	165	14.5
Stillbirth	7	0.6
Neonatal death	4	0.4

The majority of HCPs who responded to the survey were midwives (67%), with remaining respondents comprising doctors (consultant obstetricians and resident doctors), sonographers and maternity support workers. HCPs worked across a range of settings and over half had been in post for 5 years or more (online supplemental table 1). As the surveys of service users and HCPs were designed together to assess implementation and experiences of the SBLCBv2, we have organised the results by individual element.

### Element 1: CO monitoring and smoking cessation

Almost all HCPs reported implementing element 1 of the SBLCBv2 (99%). 92% of HCPs reported offering pregnant women a CO breath test and 92% of women reported taking the test (table 3). Most HCPs reported having referral pathways to smoking cessation services in place, yet only 45% of women who smoked reported being referred, with fewer than half attending their appointments (44%). Nonetheless, half of the women who smoked at the beginning of pregnancy (10%) had stopped smoking by the end of pregnancy. 16% of smokers reported switching to e-cigarettes and 21% expressed no desire to stop smoking. HCPs reported having sufficient time, CO monitors and training to carry out CO monitoring during antenatal appointments. Most staff reported recording women’s CO readings; however, a lack of time, access to computers or opting for handheld notes prevented some HCPs from doing so.

**Table 3:** Self-reported implementation of the SBLCBv2 elements

	Source	No./No. of responses	%
Element 1			
Offered carbon monoxide breath test	Service user	1046/1140	91.8
Smoked in pregnancy	Service user	111/1140	9.7
Referred to smoking cessation	Service user	50/111	45.0
Smoked at delivery	Service user	58/1140	5.1
Ceased smoking	Service user	53/111	47.7
Element 2			
Offered aspirin	Service user	295/1140	25.9
Symphysis-fundal height measured	Service user	888/1140	77.9
Suspected SGA/FGR	Service user	345/1140	30.3
Scanned due to suspected SGA/FGR	Service user	308/345	89.3
Used aspirin algorithm	HCP	267/384	69.5
Used FGR risk assessment algorithm	HCP	178/387	46.0
Element 3			
RFM leaflet before 28 weeks	Service user	713/1140	62.5
Monitored fetal movements	Service user	966/1140	96.8
Attendance for perceived RFM	Service user	584/1140	51.2
Scanned	Service user	156/584	26.7
CTG performed	Service user	526/584	90.1
Recommended delivery	Service user	47/584	8.1
RFM checklist	HCP	171/455	37.6
Element 4			
CTG in labour	Service user	508/904	56.2
Annual training CTG	HCP	299/312	95.8
Annual training in intermittent auscultation	HCP	247/312	79.2
CTG competency assessment	HCP	125/312	40.1
Intermittent auscultation competency assessment	HCP	125/312	40.1
Element 5			
Offered mid-stream urine test	Service user	794/1140	69.6
At risk of preterm birth	Service user	166/1140	14.6
Attended a preterm birth prevention clinic	Service user	36/166	21.7
Received magnesium sulfate	Service user	30/1140	2.6
Received corticosteroids	Service user	75/1140	6.6
Delivered preterm	Service user	117/1140	10.3
Preterm birth risk assessment algorithm	HCP	168/354	47.5

CTG, cardiotocography; FGR, fetal growth restriction; HCP, healthcare professional; RFM, reduced fetal movement; SBLCBv2, second version of the Saving Babies’ Lives Care Bundle; SGA, small for gestational age.

### Element 2: detection and management of FGR

70% of the HCPs surveyed reported implementing element 2. Most reported assessing women at booking to determine if aspirin should be prescribed (70% used the care bundle algorithm), with 25% of women reporting being offered aspirin (table 3) significantly higher than 11% in the ASPRE (Combined Multimarker Screening and Randomized Patient Treatment with Aspirin for Evidence-Based Preeclampsia Prevention) trial.^8^ Nearly all HCPs reported using a risk assessment pathway to triage women at increased risk of FGR into an appropriate clinical pathway; 46% used the algorithm contained in the care bundle.

Among the women who were told their baby was growing smaller than expected (30%), 89% underwent serial growth scans, with 65% of women receiving three or more scans. Most HCPs felt competent in measuring and plotting symphysis fundal height and knowing when to refer women for growth scans. However, 45% believed that some referrals were unnecessary (this differed by profession 67% of sonographers compared with 40% of doctors and 37% of midwives believed women were referred for scans unnecessarily), and 27% reported the increased demand for ultrasound scans could not always be met due to a shortage of sonographers trained in uterine artery Doppler.

### Element 3: RFM

72% of HCPs reported implementing element 3. Nearly all staff (94%) discussed RFM at every antenatal visit, and most women confirmed their midwife discussed movements at every (81%) or some (16%) antenatal appointments (table 3). Almost all staff provided RFM advice leaflets to women by 28 weeks of pregnancy, with 63% of women recalling receiving one. The majority of women reported monitoring their baby’s movements throughout pregnancy; however, the leaflet was not the primary motivator, with 67% of women acting on the advice of their healthcare provider. 43% of women said monitoring movements made them feel calm, and 47% said it made them feel anxious during pregnancy.

Among women attending maternity services with concerns about baby’s movements (51%), a large proportion received computerised cardiotocography (CTG) (90%), 27% received an ultrasound scan and 8% were recommended delivery. 85% of staff used a checklist to manage women attending with concerns about baby’s movements; 38% used the SBLCBv2 checklist. According to staff, the biggest barrier for women not attending antenatal triage for suspected RFM was a sense of burden (39%), followed by a lack of knowledge regarding the risks (18%) and cultural values (18%). Other reasons included a lack of childcare (10%), transport (8%) and time (6%).

### Element 4: intrapartum fetal heart rate monitoring

56% of the women who laboured reported receiving CTG to monitor fetal well-being and 26% reported the use of intermittent auscultation (IA) (table 3). While the majority of staff reported receiving annual training in CTG (96%) and IA (79%), only 40% reported receiving competency assessment in CTG or IA. When asked about perceived competency, most felt proficient in the use of IA and virtually all staff conveyed competence in interpreting CTG, knowing when to transition from IA to CTG, and from normal to abnormal CTG. Most staff said they knew who to escalate concerns to when the CTG is abnormal.

### Element 5: prevention and management of preterm birth

57% of the HCPs reported implementing element 5. Most staff felt competent in assessing a woman’s risk of preterm birth and stratifying them to the most suitable pathway. Almost all reported using a guideline for managing a woman’s risk of preterm birth; 47% used the risk assessment tool in the SBLCBv2 (table 3).

Of the women surveyed, 70% reported being offered a midstream urine test for asymptomatic bacteriuria, with 20% receiving treatment for a urinary tract infection. 13% of women received an ultrasound scan to measure cervical length and 2% underwent cervical cerclage. 3% received magnesium sulfate and 7% received corticosteroids.

Of the 15% of women who were identified as at risk of preterm birth (10% delivered preterm), only 21% attended a preterm birth prevention clinic. Most staff felt confident in determining when to refer women to preterm birth prevention clinics but reported lower competency in knowing when to prescribe corticosteroids and magnesium sulfate.

### Staff views of implementation

Most staff felt the care bundle interventions were clear, understandable and evidence-based, with strong support for its implementation (table 4). Staff generally reported positive experiences of implementing the SBLCBv2 and felt supported by leadership and colleagues.

**Table 4:** Staff opinions on the implementation and effects of the SBLCBv2

In your opinion	Strongly disagree	Disagree	Neither agree nor disagree	Agree	Strongly agree
The interventions are clear and understandable	24 (4%)	15 (2%)	81 (13%)	378 (60%)	135 (21%)
The care bundle is evidence based	7 (1%)	18 (3%)	99 (16%)	356 (56%)	153 (24%)
The care bundle is effective at meeting its objectives	4 (1%)	33 (5%)	186 (29%)	350 (55%)	60 (9%)
The care bundle supports my clinical decision-making	2 (0%)	17 (3%)	118 (19%)	400 (63%)	96 (15%)
I support the implementation of the care bundle	4 (1%)	5 (1%)	54 (9%)	339 (54%)	231 (36%)
I feel I provide a better level of care	8 (1%)	56 (9%)	214 (34%)	294 (46%)	61 (10%)
Workload					
The intervention(s) take too much time	35 (6%)	252 (40%)	230 (36%)	104 (16%)	12 (2%)
There is not enough staff to enable me to do my job	19 (3%)	118 (19%)	117 (18%)	257 (41%)	122 (19%)
My workload has increased	13 (2%)	97 (15%)	201 (32%)	243 (38%)	79 (12%)
I work longer hours	23 (4%)	196 (31%)	228 (36%)	135 (21%)	51 (8%)
I have less time with each patient	21 (3%)	198 (31%)	228 (36%)	141 (22%)	45 (7%)
Culture and leadership					
Leadership is driving implementation in my organisation	8 (1%)	54 (9%)	149 (24%)	320 (51%)	102 (16%)
My colleagues support the adoption of the care bundle	1 (0%)	11 (2%)	139 (22%)	403 (64%)	79 (12%)
The care bundle is now embedded in routine practice	2 (0%)	14 (2%)	133 (21%)	370 (58%)	114 (18%)
The roll-out of version 2 was communicated well	25 (4%)	116 (18%)	211 (33%)	231 (36%)	50 (8%)

In your opinion, the number of….	Greatly decreased	Slightly decreased	Not changed	Slightly increased	Greatly increased
Inductions has	10 (2%)	8 (1%)	41 (6%)	159 (25%)	415 (66%)
Caesareans has	8 (1%)	10 (2%)	57 (9%)	202 (32%)	356 (56%)
Preterm births has	15 (2%)	136 (21%)	288 (45%)	157 (25%)	37 (6%)
Stillbirths has	40 (6%)	197 (31%)	287 (45%)	89 (14%)	20 (3%)
Neonatal deaths has	45 (7%)	175 (28%)	357 (56%)	49 (8%)	7 (1%)
Babies admitted to neonatal care has	13 (2%)	120 (19%)	257 (41%)	201 (32%)	42 (7%)
Babies dying following admission to neonatal care has	45 (7%)	186 (29%)	371 (59%)	29 (5%)	2 (0%)
Incidents has	23 (4%)	128 (20%)	229 (36%)	186 (29%)	67 (11%)

SBLCBv2, second version of the Saving Babies’ Lives Care Bundle.

Staff felt the SBLCBv2 supported their clinical decision-making and that it is effective at meeting its objectives; however, some were concerned around staff shortages (22% of respondents) and communication gaps regarding its rollout (22%). There was no consensus whether staff felt they were working longer hours or spending less time with patients. 90% of staff supported the roll-out of SBLCBv2 in their organisation.

Staff perceived increases in inductions of labour and caesarean sections over the preceding 5 years. Opinions on stillbirths, neonatal deaths and preterm births varied, with some staff believing they had decreased while others thought they had remained unchanged. Similarly, staff felt that admissions to the neonatal unit had either increased or had not changed, with a notable number perceiving increases in the number of incidents.

### Women’s views of maternity care

#### Antenatal experience

A total of 1071 women’s written responses about their antenatal care were analysed, of which 89% reported a positive experience. Three themes summarised women’s positive or negative antenatal experiences in pregnancy: (1) feeling listened to and reassured by care, (2) feeling in control of decision-making and (3) encounters with staff and care. Supporting quotes are in table 5.

**Table 5:** Quotes for the three themes, examples from women reporting positive and negative experiences of antenatal care informed by the SBLCBv2

Positive examples		Negative examples
*Theme 1: Feeling listened to and reassured by care*		
*‘Great communication, always felt like I was being listened to and given the best advice’ (Important Principles*) *‘During my pregnancy I felt like I was kept informed and any concerns I had were dealt with thoroughly and with care and empathy. I felt like the hospital and community teams communicated well and the additional screening for preeclampsia and the additional 36-week scan showed an enhanced level of care’ (Important Principles*)	***Subtheme 1: Communication***	*“I felt really dismissed by the midwives when I kept saying the baby wasn’t moving and I didn’t feel well” (Element 3*) *“Also nothing was consistent, I’d be told one thing at hospital by a midwife by a consultant and then my community midwife would say something else again.” (Important principles*)
*‘All testing done efficiently, staff were continually informing us about the decisions they were making, we felt well taken care of’ (Important principles*)	***Subtheme 2: Medical intervention***	*“While yes I got growth scans nothing was ever explained and I wasn’t able to ask any questions” (Element 2*)
*‘I felt listened to, I had the same midwife for most of my appointments which helped with continuity and a relationship.’ (Important principles*)	***Subtheme 3: Tailored care***	*“As a third time Mum, it didn't really phase me but I imagine as a first time Mum, I wouldn't have felt well cared for with so many different people involved. No consistency to build a rapport up” (Important principles*)
*Theme 2: feeling in control of decision-making*		
*‘The staff were attentive and provided me with all the information I required. This was offered and also given 24 hours a day with no bias.’ (Important principles*)	***Subtheme 1: Being informed***	*“Quality of advice was sometimes lacking. Had to rely on own research.”*
*‘Seen for growth scans due to BMI. All appointments were made in advance for diabetes test, 20 week anatomy scan, week 32, 35, 38 all made at one appointment and text/email appointments which was great to be able to let work know in advance…’ (Element 2*)	***Subtheme 2: Timely and accessible appointments***	*“I was gestational diabetic, with reduced fetal growth and movement and border line cholestasis. Near the back end of my pregnancy (when I wanted to discuss induction as an option). Consultant appointments were over subscribed. I couldn’t see the diabetic team or a consultant which was highly worrying” (Element 2*)
*Theme 3: Encounters with staff and care.*		
*‘I was made to feel like nothing was too much trouble during every step of my pregnancy, the staff were always so lovely, happy and welcoming even when you could see they were so busy’ (Important principles*)	***Subtheme 1: Encounters with staff***	*“The staff on reception were very rude and never welcoming. The sonographers some were lovely and explained a lot in detail, others were not so talkative and huffed every time we asked a question’ “Element 2 and Important Principles”*
*‘‘All services have been very supportive and understanding, they have been very reassuring with any worries or concerns’. (Important principles*)	***Subtheme 2: Encounters with care***	*“The administration side of things was not great. I was forgotten about on numerous occasions. Letters were sent out with appointment dates that didn’t exist. I would turn up and people weren’t expecting me.” (Important principles*)

SBLCBv2, second version of the Saving Babies’ Lives Care Bundle.

#### Theme 1: feeling listened to and reassured by care

Women who reported a positive antenatal experience felt listened to by staff which fostered a sense of reassurance. Negative experiences were associated with not feeling listened to and dismissal. Three subthemes defined this theme: (1) communication, (2) medical intervention and (3) tailored care (table 5).

##### Subtheme 1: communication by staff

Communication was central to women feeling listened to and positive experiences saw staff acknowledge their history and women feeling able to ask questions. Whereas, poor communication with staff led to women feeling dismissed; an experience that was particularly common when expressing concerns about RFM. Poor communication between staff made care feel disjointed and was further hindered with the change to electronic notes.

##### Subtheme 2: medical intervention

Women felt reassured by effective testing, monitoring and the offer of frequent scans; however, when this offering was not well explained, it resulted in reported errors in care and women feeling confused about the reasons for the intervention.

##### Subtheme 3: tailored care

Tailored care through a specialist care pathway (e.g., mental health support) or continuity of carer enhanced the antenatal experience. Continuity of carer ensured care felt more personal and safer, whereas having multiple care providers saw conflicting information being given, care-related errors being made and women found it more difficult to ask sensitive questions due to a lack of trust and rapport.

### Theme 2: feeling in control of decision-making

Feeling well informed about decisions during pregnancy made women feel in control of all aspects of their pregnancy. A negative antenatal experience was reported when information was not given to women. Two subthemes were evident: (1) being informed and (2) timely and accessible appointments.

#### Subtheme 1: being informed by staff

Women felt empowered when staff provided comprehensive information of all the options available to them and supported women to have autonomy over their decisions. A lack of information provided by staff made women feel less able to make informed choices and pressured into decisions.

#### Subtheme 2: timely and accessible appointments

Timely and accessible appointments provided time and space for women to discuss concerns and management options which helped reduce anxiety and support decision-making. Extra appointments and scans due to their history were well received, but women were left feeling anxious and angry when appointments felt rushed, were a long distance away, had long wait times or were cancelled.

### Theme 3: encounters with staff and care

Finally, women commented on their encounters with staff and care. Examples tended to be a mass of adjectives with little detail about the encounters.

#### Subtheme 1: examples of encounters with staff

Positive encounters included words such as caring, professional, thoughtful, calming, sympathetic, reliable, compassionate, understanding, kind, knowledgeable, professional, helpful, non-judgemental and patient. Negative encounters with staff included being rude, unhelpful, unsupportive, uncompassion and judgemental.

#### Subtheme 2: examples of encounters with care

Positive encounters with care were reported as smooth, efficient and helpful. Examples of negative encounters with health hospital care included delays in care, unnecessary appointments, limited services and poor organisation.

### Labour and birth experience

A total of 1044 women’s responses about labour and birth care were analysed and reflected positive perceptions by 86% of women. IOL was mentioned by 147 women, of which 71% reported a positive experience. Three themes summarised the women’s labour and birth experiences: (1) attitudes and behaviours of staff led to a supported and safe labour and birth, (2) good communication made women feel informed and respected and (3) a broken care system (table 6).

**Table 6:** Quotes for the three themes, examples from women reporting positive and negative experiences of care during labour and birth informed by SBLCBv2

Positive examples		Negative examples
*Theme 1: Attitudes and behaviours of staff lead to a supported and safe labour and birth*		
*“The staff on maternity and delivery were amazing. I can’t thank them enough for the care we received. In particular they were all fully aware of previous birth trauma I’d experienced and did everything they could to provide reassurance and support and tried everything to make sure I didn’t sustain birth injury or trauma as I did last time.” (Important principles*)	***Subtheme 1: Reassurance made them feel supported.***	*“I was judged because I was young and they made me feel like an inconvenience when I went in for reduced movements. My waters had gone and been gone for a few days and they didn’t believe me.” (Element 3*)
*“I was amazed with the staff and ways of working. Everyone was super helpful and friendly. I had a midwife and trainee midwife so always had two people in the room at all times. Everything they did they explained and answered any questions I had. I felt they were very attentive and caring having mine and the baby’s best interest at heart.” (Element 4, Important principles*)	***Subtheme 2: Felt safe and cared for by staff***	*“During the days up to my baby being born I had a different pattern of movement which changed significantly, I went in again with reduced movement and the team agreed and give me the option to be induced. I took this option. The consultant was very rude and came the day after and said she would not have given me this option if the midwives had asked her specifically as there was still movement, she told me I was being dramatic and I just wanted to hurry the pregnancy up(…). She was unprofessional, unhelpful and rude. I dreaded seeing her again as I was made to feel silly.” (Element 3*)
NA	***Subtheme 3: Lack of person-centred care***	*“My birth plan was the most natural possible. It was my fourth and last pregnancy. I wanted it to be a good memory like the others(…)They did what they wanted, they didn’t listen to me, ie,nded up with a c-section for no reason. I’ve still not recovered mentally from what happened. (Important principles*)
*Theme 2: Good communication made them feel informed and respected*		
*“The staff respected my birth preferences, even when some of it went against their recommendations. They mostly left us to it which was great for me to get in the zone. Staff acted quickly when need be and remained professional although clearly a very busy department.”* *“Understanding, caring staff who kept me informed at all times and were there when I wanted to ask a question or needed advice. Fantastic midwives and doctors who delivered my baby girl.” (Element 4, Important principles*)	***Subtheme 1: Feeling informed***	*“My labour halted at 9cm. I was offered a drip of oxytocin. The side effects weren’t really explained. This did not progress things therefore I needed to have an emergency c-section. We asked earlier what would be the implications of this but were told ' we will worry about it if it happens’. By the time it happened I was in too much pain to understand anything. I don't believe in this wait and see attitude.” (Important principles*)
*“I was induced to do reduced movements, during the induction process including a midwife breaking my waters, they were friendly and I was fully informed of everything that was happening and all my options of pain relief.” (Element 3, Important principles*)	***Subtheme 2: wishes and concerns were listened to and respected***	*“Pressure to induce for no specific to me reason other than check boxes from 39 weeks and continuous pressure each time I turned down induction (3x). Eventually told my baby had stopped growing and had been static for a month and everyday I turned down induction I was ‘one day closer to a still born baby’ (40+8) of course at this point I was feeling so guilty and worried and so I agreed to be induced.” (Element 2, Important principles*)
*Theme 3: Broken care system*		
*NA*	***Subtheme 1: Delays and absence of care***	*“The induction process is a frustrating journey of not knowing what will happen or how long you will be in hospital or if you will have a delivery similar to the one you imagined.” (Induction of labour may be recommended in element 2 or 3*).
*NA*	***Subtheme 2: Errors in care***	*“My son died 7 hours after birth due to lack of oxygen during labour. This was due to the midwife in charge of my care not following guidelines or doing the correct checks and monitoring. From the moment I walked into the ward I felt as though I was an inconvenience and didn’t feel comfortable or cared for at all. I also didn’t get the correct pain relief when my episiotomy and forceps delivery occurred which resulted in extreme pain and extra trauma (obviously not as traumatic as my son dying in front of me).” Element 4*)

SBLCBv2, second version of the Saving Babies’ Lives Care Bundle.

#### Theme 1: attitudes and behaviours of staff lead to a supported and safe labour and birth

Midwives were mentioned most by women as playing a vital role in labour and birth experiences. The attitudes and behaviours displayed by staff were reported as key to a positive labour and birth experience. Similarly to antenatal care, attentive, compassionate and friendly staff were described in positive responses, whereas negative experiences were attributed to poor attitudes and behaviours of staff and led to women feeling judged, including staff being judgemental, disinterested and unempathetic. Three subthemes explain how these attitudes and behaviours of staff impacted on women’s experience: (1) reassurance made them feel supported, (2) feeling safe and cared for by staff and (3) lack of person-centred care.

##### Subtheme 1: reassurance made them feel supported

The support offered by staff, namely midwives, offered reassurance to women which made their labour and birth journey a positive one. Central to feeling reassured was their knowledge and understanding of individual pregnancy histories and emotional reassurance throughout the process of labour. Some women reported not feeling supported or reassured as staff did not respond to their needs in relation to maternal perception of RFM. Reports of conflicting responses from staff about induction decisions were also reported.

##### Subtheme 2: felt safe and cared for by staff

Women felt safe and cared for because of the care provided by staff during labour and birth. A key component of this care was feeling listened to by staff. Not feeling listened to by staff was mentioned in reported negative experiences, many of which involved RFM and reporting pain.

##### Subtheme 3: lack of person-centred care

Several women who had a negative experience reported feeling that staff treated them in a way that left them feeling that they were not central to their care and instead felt humiliated, like an inconvenience, a hindrance or like an object.

### Theme 2: good communication made women feel informed and respected

Good communication between women and staff in the changing situation of labour and birth was mentioned positively by women when staff were responsive while ensuring women were informed and their choices respected. Where communication was lacking or poor, women reported a negative experience with incorrect or inconsistent information being given to them. Two subthemes demonstrate why good communication was important to women and enhanced their labour and birth experience: (1) feeling informed and (2) wishes and concerns were listened to and respected.

#### Subtheme 1: feeling informed

Women who had a positive experience felt informed about intervention(s) and choices that arose during their pregnancy. When staff gave women relevant and timely information, they felt supported and able to make informed decisions. When birth plans could not be followed, women were happy when they were informed of the reasons why. However, there were instances when information regarding rationale for, and the risks and benefits of, interventions was lacking.

#### Subtheme 2: wishes and concerns were listened to and respected

Women who had a positive experience of labour and birth felt they were listened to by staff, especially midwives, which in turn meant they felt their choices were respected. However, some women felt dismissed and thus did not feel supported by staff in relation to choices about labour. Most women felt induction was a choice that was fully explained to them, but a minority felt pressured into an IOL.

### Theme 3: a broken care system

This theme was only reported by women expressing a negative experience of labour and birth. Many examples of poor care were reported by women, including disorganised and inconsistent care, staff shortages, lack of beds and poor pain management. Two subthemes give more detail: (1) delays and absence of care and (2) errors in care.

#### Subtheme 1: delays and absence of care

Women who had a negative labour or birth experience reported delays or absence of care which left them feeling neglected. This included delayed and failed inductions or a lack of pain management.

#### Subtheme 2: errors in care

Errors in care were reported by women who had a negative experience of labour and birth. These include errors in medical procedures, missed diagnoses, rushed procedures and serious injuries.

## Discussion

This study demonstrates that interventions included within the SBLCBv2 have continued to be integrated into maternity care; however, the degree of implementation between elements remains variable. Women’s experiences of care were positively influenced by principles laid down in SBLCBv2 specifically offering choice and personalised care, promotion of continuity of carer and providing information to maternity service users. Conversely, the lack of these elements of care contributed to negative experiences of care. It is important to acknowledge the importance of good communication between HCPs and service users to address the possible tension between provision of standardised care pathways against personalised care.

Triangulating data from other national sources supports the observation that some elements are better implemented than others. The Perinatal Mortality Review Tool, used to review all perinatal deaths, indicates that between 2018 and 2023 the proportion of cases with issues relating to assessment or management of smoking cessation fell from 13% to 5%, those with issues relating to inadequate fetal growth surveillance fell from 30% to 21% though investigation and management of RFM has remained constant at approximately 16% of cases.^9^ Although not directly comparable, the Care Quality Commission national maternity survey indicated that 76% of respondents received information about warning signs related to pregnancy complications and 83% said their midwife always listened to them.^10^ However, a significantly lower proportion said they were involved in the decision for IOL (59%). These experiences are reflected in participants’ about their experiences of care, and in our linked interview study.^10^

There is some evidence that care bundles reduce the risk of negative outcomes,^11^ but the evidence for the effectiveness of care bundles in maternity care comes from a small number of studies.^312^,^14^ Evaluation of PeriPREM, an 11-item care bundle, in 12 maternity units increased implementation from 3% to 29% over time, but only some of the individual components’ implementation significantly increased.^13^ Importantly, this study found that implementation was associated with staff reporting improved team function, situation monitoring and communication with teams.^13^ Implementation of some components of SBLCBv2 also increased over time, indicating ongoing adoption into routine care. Where comparable metrics were available,^3^ we found a greater proportion of service users (than in 2017) were offered a CO test, though fewer smokers were referred to smoking cessation services and a higher proportion stopped smoking (online supplemental table 2). In element 3, fewer women received a leaflet about fetal movements, a greater proportion of women attended with RFM, significantly more had a fetal heart rate trace and significantly less had IOL following RFM.

A realist evaluation of implementation of the preterm birth element (element 5) of SBLCBv2 (IMplementation of the Preterm Birth Surveillance PAthway: a RealisT evaluation (IMPART) study) in three maternity units identified several barriers to effective implementation including a lack of knowledge about risk factors for complications and purpose of the pathway, and need for adequate resources.^14^ Critically, in our evaluation, the majority of staff felt the care bundle supported decision-making, but some felt the SBLCBv2 interventions took too much time and there were insufficient staff to deliver them. In addition, the IMPART study also noted a third theme of woman-centred care highlighting the value of prior knowledge of the service user and need for clinicians to have time and freedom to provide flexible and individualised care.^14^ This latter point echoes findings from other service-users deemed to be high-risk for complications (women with obesity and those with gestational diabetes).^15 16^ The importance of risk factors was not always communicated effectively and women were often given no choice in their treatment, which meant they felt threatened or frustrated. There was an undue focus on fetal well-being without consideration of the impact on women. The points are all reflected in the service-users’ comments about antenatal and intrapartum care influenced by the SBLCBv2 in this study. Service users need to feel heard, involved in and reassured by their care. To this end, the communication between HCPs and service users is critical, and behaviours of staff convey attitudes. These findings suggest that care directed by SBLCBv2 could be improved through education of HCPs about risk factors for preterm birth and stillbirth and how the SBLCBv2 addresses these pathways. This knowledge will need to be combined with effective communication skills to ensure that service users receive information to make an informed choice, ensuring they retain agency and perceived control. This will enable the core recommendations of SBLCBv2 to be personalised to individual service users, promoting safe maternity care and improved maternity experience.

This study was strengthened by being conducted according to a published protocol including both service users’ and professionals’ views. We were able to reach a range of professionals including midwives, obstetricians, maternity support workers and sonographers, all of whom have roles in implementing SBLCBv2. However, this study was limited by an inability to calculate the response rate as we used a variety of approaches (social media, circular emails) to reach service users and staff, thus the denominator is unknown. While we sampled service users from different ethnic groups, our sample was biased with 86% coming from white backgrounds compared with 61% of births. Given the ongoing disparity in stillbirth rates in Asian and black women,^17^ purposive sampling of women from these groups is needed to determine the degree of SBLCBv2 implementation in these populations and identify areas which can be targeted for improvement.

Like the IMPART study, we initially intended to use routinely collected data to assess fidelity of SBLCBv2 implementation; however, these data were of inadequate quality to achieve this. We have attempted to triangulate our data from other sources to ensure validity of our conclusions. However, prospectively designed collection of standardised data on process and outcome measures is essential for ongoing evaluation of national initiatives designed to improve outcomes for mothers and babies.^18^ Specific focus is needed to determine whether the SBLCB or approaches to its’ implementation needs to be adapted to meet the needs of women from non-white ethnic groups. Optimally, planning for evaluation would occur during the intervention design such that its impact and barriers and facilitators to success can be appreciated.

Contemporary antepartum and intrapartum care appears to have been influenced by the SBLCBv2. By inference, implementation of the SBLCBv2 will impact on service users’ experience of maternity care. However, SBLCBv2 has not been implemented in isolation; its launch coincided with the COVID-19 pandemic and parallel initiatives including PeriPREM and learning generated from investigations and reviews of perinatal deaths will also have influenced care. Thus, this evaluation can only report level of implementation of elements of SBLCBv2 at a point in time and cannot definitely conclude improvements in outcomes or experience result from SBLCBv2 implementation. Nevertheless, our data suggest that elements of the SBLCBv2 are increasingly embedded in maternity care, but refinements are still needed to address variation in practice between units and to support effective communication between HCPs and service users to balance standardised clinical practice with personalised care.

## Supplementary material

10.1136/bmjoq-2025-003456 online supplemental file 1

10.1136/bmjoq-2025-003456 online supplemental file 2

10.1136/bmjoq-2025-003456 online supplemental file 3

## Data Availability

Data are available on reasonable request.
