# Supplementary material for: Evaluating the implementation of the Saving Babies Lives Care Bundle Version 2 from service user and healthcare professionals’ perspectives: a questionnaire study
Source: BMJ Open Qual. 2025 Sep 2;14(3):e003456. doi: 10.1136/bmjoq-2025-003456 (PMC12410671; doi:10.1136/bmjoq-2025-003456)
Supplement: online supplemental file 1 [file bmjoq-14-3-s001.docx]

**Supplementary Table 1** - Characteristics of the 633 healthcare professionals responding to the survey

| Role | No. Responses | % |
| --- | --- | --- |
| Midwife | 430 | 67.9 |
| Doctor | 123 | 19.4 |
| Maternity support worker | 35 | 5.5 |
| Sonographer | 33 | 5.2 |
| Other | 24 | 3.8 |
| Work setting |  |  |
| Labour ward or birth centre | 320 | 50.6 |
| Antenatal clinic | 228 | 36.0 |
| Postnatal ward | 227 | 35.9 |
| Maternity assessment or triage | 201 | 31.8 |
| Antenatal ward | 195 | 30.8 |
| Community | 125 | 19.7 |
| Early pregnancy unit | 83 | 13.1 |
| Ultrasound department | 45 | 7.1 |
| Practice education | 42 | 6.6 |
| Fetal medicine department | 36 | 5.7 |
| Neonatal unit | 8 | 1.3 |
| Other | 74 | 11.7 |
| Years at organisation |  |  |
| More than 15 years | 156 | 24.6 |
| 11-15 years | 76 | 12.0 |
| 6-10 years | 128 | 20.2 |
| 3-5 years | 111 | 17.5 |
| 1-2 years | 92 | 14.5 |
| Less than 1 year | 70 | 11.0 |
