# Supplementary material for: Evaluating the implementation of the Saving Babies Lives Care Bundle Version 2 from service user and healthcare professionals’ perspectives: a questionnaire study
Source: BMJ Open Qual. 2025 Sep 2;14(3):e003456. doi: 10.1136/bmjoq-2025-003456 (PMC12410671; doi:10.1136/bmjoq-2025-003456)
Supplement: online supplemental file 2 [file bmjoq-14-3-s002.docx]

**Supplementary Table 2** – Comparison between implementation of elements of the SBLCB and process measures in the evaluations of implementation of Version 1 (SPiRE) and Version 2 (eVOLVE).

| **SBLCB Element** | **Component** | **Implementation in SPiRE** | **Implementation in eVOLVE** | **Trend** |
| --- | --- | --- | --- | --- |
| Element 1 | Service users offered carbon monoxide testing | 70.1% | 91.8% | ↑ |
|  | Service users who smoke referred to smoking cessation service | 60.1% | 45.0% | ↓ |
|  | Service users who smoked at booking who ceased smoking | 40.8% | 47.7% | ↔ |
| Element 2 | No directly comparable assessments | | |  |
| Element 3 | Proportion of service users given leaflet on reduced fetal movement (RFM) | 74.4% | 62.5% | ↓ |
|  | Proportion of service users attending for RFM | 36.5% | 51.2% | ↑ |
|  | Proportion of service users attending with RFM who had an ultrasound scan | 29.4% | 26.7% | ↔ |
|  | Proportion of service users attending with RFM who had a fetal heart rate trace | 73.5% | 90.1% | ↑ |
|  | Proportion of service users who had induction of labour following RFM | 54.7% | 8.1% | ↓ |
| Element 4 | No directly comparable assessments | | |  |
