# Supplementary material for: Evaluating the implementation of the Saving Babies Lives Care Bundle Version 2 from service user and healthcare professionals’ perspectives: a questionnaire study
Source: BMJ Open Qual. 2025 Sep 2;14(3):e003456. doi: 10.1136/bmjoq-2025-003456 (PMC12410671; doi:10.1136/bmjoq-2025-003456)
Supplement: online supplemental file 3 [file bmjoq-14-3-s003.docx]

**Supplementary File**

Questionnaires used for survey of Maternity Service Users (Pages 2-20) and Health Care Professionals (Pages 21-41).


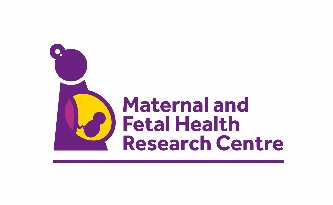

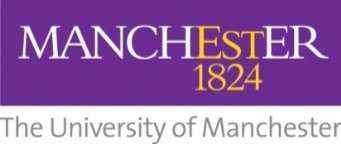

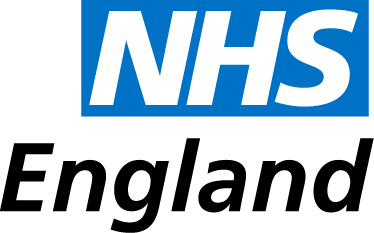


**Maternity Care Survey**

**What is this survey about?**

You are invited to take part in a survey being carried out by the University of Manchester on behalf of NHS England. The survey asks about your **most recent** experience of maternity care. **Your views are very important to us.** They help us to understand more about the quality of NHS maternity care services across England and what needs to be improved.

Taking part is **voluntary** and **anonymous.** None of the healthcare professionals involved in your care will know whether or not you have taken part. Choosing to take part will not disadvantage you in anyway.

**Am I eligible to take part?**

If you are aged **16 years** **+**, **gave birth in the last 12 months**, and can **understand/speak English**, you are eligible to take part.

**What do I need to do?**

You will be asked to complete an online survey. It should take approximately 10-20 minutes to complete. Firstly, you will be asked to confirm your eligibility. The survey will then ask you about your antenatal care, labour and birth. Finally, it will ask you questions about yourself, such as age and ethnicity.

Once you have completed the survey, you will be given the option to provide your contact details if you are interested in taking part in a short follow-up interview and would like the researchers to contact you. Contact details will be kept strictly confidential and stored separately to your survey responses. You do not have to provide your contact details if you do not want to.

**Can I withdraw from the study?**

Because your responses are anonymous, once you submit your responses, you will not be able to withdraw them. Thus, by completing the survey you understand your answers cannot be withdrawn and will be used in this study.

If you provide your contact details, we will review your surveys responses in order to select certain groups of women to interview. This is so we can ensure we have heard the views of a wide range of women from different backgrounds. This does not affect your survey responses which will be analysed anonymously.

**What are the risks of taking part?**

We understand that some questions may be upsetting if you have experienced a negative pregnancy / birthing experience. If you are feeling distressed we recommend you pause the survey, come back to it another time or stop altogether. Should you experience distress, we recommend you contact the research team using the contact details at the end of this sheet or the following organisations for emotional support.

**Maternal Mental Health Alliance**: For a variety of links to support and resources for mothers and families, please visit this webpage: [https://maternalmentalhealthalliance.org/resources/mums-and-families/](about:blank)

**NHS Webpage:** The following website contains several services and support for new parents: [https://www.nhs.uk/conditions/pregnancy-and-baby/services-support-for-parents/](about:blank)

**Family Lives**: Confidential support, information and advice for new parents and families, you can visit: [http://www.familylives.org.uk/](about:blank)

**Tommy’s:** for support for mothers and families who have experienced baby loss: [www.tommys.org](http://www.tommys.org/)

**Samaritans (emotional support):** 116 123

**What will happen to the information I supply?**

Your responses will be analysed by the research team at the University of Manchester. The results will be published on the NHS England website at the end of the study. Once the study has ended we will keep your answers for 5 years in line with the University of Manchester’s recommendations. The information may be looked at by responsible individuals at the University of Manchester and regulatory authorities. Any contact details you supply will only be kept until the end of the study and then destroyed. We will not pass them on to any third parties. Anonymous survey responses will be permanently stored in the University’s data repository.

**Can I get more information about the study?**

If you have any queries about the study or have any questions please contact the research team at the University of Manchester or your healthcare professional.

Kate Widdows: [Kate.Widdows@manchester.ac.uk](mailto:Kate.Widdows@manchester.ac.uk)

Debbie Smith: [Debbie.Smith-2@manchester.ac.uk](mailto:Debbie.Smith-2@manchester.ac.uk)

**You do not have to complete the questionnaire if you do not want to.**

**Eligibility questions**

1. Are you 16 years or over?

a Yes

b No

*If b, “You are not eligible to take part in this study. Thank you for your time in considering this study. Please close your browser to exit the survey”.*

1. When did you give birth?

a May 2022

b June 2022

c July 2022

d August 2022

e September 2022

f October 2022

g November 2022

h December 2022

i January 2023

j February 2023

k March 2023

l April 2023

m May 2023

n June 2023

o July 2023

p August 2023

q September 2023

r October 2023

1. If you are happy to proceed, please confirm you have read the previous information and agree to participate below

a Yes, I agree to participate

b No, I do not agree to participate

*If b,* **“***Thank you for your time in considering this study. Please close your browser to exit the survey”.*

# Section A - Care during your pregnancy

Thank you for agreeing to take part in this survey.

Firstly, we would like to ask you some questions about your last pregnancy. If you are currently pregnant, please tell us about your previous birth.

1. How did you hear about this survey?

a Whilst in hospital (poster or leaflet)

b From my community midwife (leaflet)

c Social media / website

d A midwife contacted me directly about the study

d Other (please enter details)

1. Which Trust provided your antenatal care?

Drop down list of Trusts

1. Which Trust provided your labour and birth?

Drop down list of Trusts

1. Overall, how do you feel about the antenatal care you received?

a Very positive

b Quite positive

c Slightly positive

d Neither positive nor negative

e Slightly negative

f Quite negative

g Very negative

1. Why did you feel this way?

Free text

1. Overall, I feel my experience of labour and birth was….

a Excellent

b Good

c Fair

d Poor

e Very poor

1. Why did you feel this way?

Free text

1. Is this your first pregnancy?

a Yes

b No

*If a, skip to question 13*

*If b, display question 12*

1. In your previous pregnancies, did you experience any of the following?

*Tick all that apply*

a Previous preterm prelabour rupture of membrane (PPROM)

b Previous use of cervical cerclage (stitch)

c Baby was small (such as fetal growth restriction or small for gestational age)

d Preterm birth (before 37 weeks of pregnancy)

e Late miscarriage (between 14 and 34 weeks of pregnancy)

f Stillbirth (baby died before birth)

g Baby died shortly after birth (within 4 weeks of life)

h Baby died within the first year of life

i None of the above

*If a, b, d or e, display question 50-51*

1. In your most recent pregnancy, did you give birth to a single baby, twins or more?

a A single baby

b Twins

c Triplets, quads or more

1. Did you have any of the following medical conditions in your most recent pregnancy?

*Tick all that apply*

a Hypertension

b Type 1 or type 2 diabetes or diabetes in pregnancy

c Pre-eclampsia

d None of the above

1. Was your booking appointment before 10+6 weeks?

a Yes

b No

c Don't remember

1. Were you offered a midstream urine test (MSU) to screen for a urinary tract infection (UTI)?

a Yes, at my booking appointment

b Yes, at multiple appointments

c No

d Don't remember

*If a or b, display question 17*

*If c or d, skip to question 18*

1. Did you receive treatment for a urinary tract infection (UTI)?

a Yes

b No

c Don't know / can't remember

# Section B – Stopping smoking during pregnancy

We would like to know about the information you received about reducing smoking in pregnancy:

During your antenatal check-ups:

1. Were you offered a breath test for carbon monoxide (CO) exposure?

a Yes, I took the test

b Yes, but I declined the test

c No, I wasn't offered the test

d Don't remember

*If a, display questions 19 and 20*

*If b, skip to question 20*

1. When were you offered the carbon monoxide (CO) breath test?

*Tick all that apply*

a At my booking appointment

b At my 36 week appointment

c On multiple occasions

d Don't remember

1. Did the test show you had high levels (above 4ppm) of carbon monoxide?

a Yes

b No

c Don’t remember

1. Did you smoke at the start of your last pregnancy?

a Yes, cigarettes

b Yes, e-cigarettes (vaping)

c Yes, cigarettes and e-cigarettes (vaping)

d No

*If d, skip to question 27*

1. Did you smoke at the end of your last pregnancy?

a Yes, cigarettes

b Yes, e-cigarettes (vaping)

c Yes, cigarettes and e-cigarettes (vaping)

d No

*If d, skip to question 27*

1. Were you referred to a stop smoking specialist?

a Yes

b No

c Don't remember

*If a, display question 24*

1. Did you attend the stop smoking service?

a Yes, I stopped smoking

b Yes, but I continued to smoke

c No I didn’t attend

*If a or b, display question 25-26*

1. Roughly how many weeks were you when you stopped smoking?

a Before 16 weeks

b Between 16 and 24 weeks

c After 24 weeks

d Not sure

1. Do you feel you received enough support by your antenatal care team to stop smoking in pregnancy?

a Yes, definitely

b Yes, to some extent

c No, I would have liked more support

d I did not want to stop smoking

e Not sure

# Section C - Checking your baby's growth

We would like to know more about how your baby’s growth was monitored during your most recent pregnancy:

1. Were you offered a prescription for aspirin?

a Yes

b No

c Can't remember/not sure

*If a, display question 28*

1. How often did you take aspirin?

a Everyday

b Sometimes

c Rarely

d Never

e Can't remember

1. Did your midwife measure your tummy using a tape measure to assess your baby's growth (known as symphysis fundal height)?

a Yes, at every appointment

b Yes, at some appointments

c No

d Can't remember/not sure

1. Were you told your baby was growing smaller than expected?

a Yes

b No

c Don't know

*If a, display questions 31-34*

*If b or c, skip to question 35*

1. At what gestation were you first told that your baby is growing smaller than expected?

a Before 28 weeks gestation

b Between 28 and 34 weeks gestation

c After 34 weeks gestation

d Can't remember

1. Did you receive any of the following?

*Tick all that apply*

a Additional ultrasound scans from 28 weeks to monitor baby’s wellbeing

b Additional ultrasound scans from 32 weeks to monitor baby’s wellbeing

c Appointment with fetal medicine

d None of the above

*If d, skip to question 35*

1. How often did you have the additional growth scans?

a Every week

b Every 2 weeks

c Every 4 weeks

d More than 4 weeks apart

e Don’t remember

1. Roughly how many additional scans did you have to monitor baby’s wellbeing?

a 1-2

b 3-4

c 4-5

d >5

e Don’t remember

1. Did you have an induced labour?

a Yes, before 37 weeks

b Yes, between 37-39 weeks

c Yes, after 39 weeks

d No

*If a, display question 36*

*If d, skip to question 37*

1. Was this decision to have an induced labour discussed with you?

a Yes

b No

c Don't remember

1. Did your baby have an abnormality diagnosed on ultrasound scan (e.g. a problem with its brain, heart or kidneys)?

a Yes

b No

# Section D - Monitoring your baby's movements

We would like to know about your experience of monitoring your baby’s movements and how they were monitored:

During your most recent pregnancy:

1. Did your midwife discuss your baby’s movements with you and what to do if they changed (reduced fetal movements)?

a Yes, at every antenatal appointment

b Yes, at some appointments

c No

d Don't remember

1. Did you receive an advice leaflet (including one that was online) on reduced fetal movement before you were 28 weeks pregnant?

a Yes

b No

c Don't remember

1. Did you monitor your baby’s movements?

a Yes, throughout my pregnancy

b Yes, through most of my pregnancy

c Yes, through some of my pregnancy

d No

*If a-c, display questions 41-42*

*If d, skip to question 43*

1. What made you decide to monitor your baby's movements?

*Tick all that apply*

a A leaflet on reduced fetal movements

b Advice from a midwife or doctor

c Advice from the internet

d Advice from family and friends

e My own experience

1. How did monitoring your baby’s movements make you feel during pregnancy?

a Very calm

b Slightly calm

c Had no effect

d Slightly anxious

e Very anxious

1. Did you attend the hospital with concerns about changes in your baby's movements after 28 weeks of pregnancy?

a Yes, I went to hospital once

b Yes, I went to hospital on two or more occasions

c No, I did not attend hospital

*If a or b, display question 44*

*If no, skip to question 45*

1. What happened when you visited the maternity unit?

*Tick all that apply*

a Listened to the baby’s heartbeat

b Electronic fetal monitoring (also known as a CTG)

c Performed an ultrasound scan

d Recommended delivery

e No action was taken

# Section E – About your labour and birth experience

Please tell us more about your labour and birth experience in your last pregnancy:

1. Were you told that your baby/babies were at risk of being born early (pre-term)?

a Yes

b No

c Don’t know

1. How many weeks pregnant were you when your baby was born?

a Before 24 weeks pregnant

b When I was 25-28 weeks pregnant

c When I was 29-36 weeks pregnant

d When I was 37 weeks or more

*If d, skip to question 50*

1. Did you attend a preterm birth prevention clinic?

a Yes

b No

1. Before you gave birth did you receive steroid injections for your baby’s lungs?

a Yes, within two days (48 hours) before I gave birth

b Yes, within one week (7 days) before I gave birth

c Yes, two weeks or more before I gave birth

d Yes, though not sure when this was

e No

f Don't remember

1. Did you receive magnesium sulphate (a drug given through your veins)?

a Yes, one day (24 hours) before I gave birth

b Yes, two or more days before I gave birth

c Yes, though not sure when this was

d No

e Don't remember

1. Were you offered an ultrasound scan to measure the length of the cervix (neck of the womb)?

a Yes

b No

c Don't remember

1. Did you have a stitch placed around your cervix to prevent preterm birth (sometimes called a cervical cerclage)?

a Yes

b No

1. During labour, did doctors monitor your baby's heartbeat?

a Yes, at regular intervals (intermittent auscultation)

b Yes, continuously (electronic fetal monitoring)

c No

d Don't know

1. What type of birth did you have?

a A vaginal birth (no forceps or ventouse suction cup)

b An assisted vaginal birth (e.g. with forceps or ventouse suction cup)

c A planned caesarean birth

d An emergency caesarean birth

1. What happened after the birth of your baby?

a My baby was well and came home with me

b My baby was admitted to neonatal care

c My baby died before birth (stillborn)

d My baby died after they were born

*If b, display question 55*

1. Was your baby's neonatal care provided in the same hospital where you gave birth?

a Yes, the same hospital

b No, my baby was transferred to a different hospital

# Section F – About you

Finally, we would now like to ask you a few questions about yourself. This is so we can ensure we have heard the views of a wide range of women from different backgrounds.

1. What is your age group?

a 16 - 18 years old

b 19 - 24 years old

c 25 - 29 years old

d 30 - 34 years old

e 35 - 39 years old

f 40+ years old

1. What best describes your ethnic group?

White

a British

b Irish

c Other

Mixed

d White and Black Caribbean

e White and Black African

f White and Asian

g Other

Asian or Asian British

h Indian

i Pakistani

j Bangladeshi

k Other

Black or Black British

l Caribbean

m African

n Other

Other ethnic groups

o Chinese

p Other

q Prefer not to say

1. Were you born in the UK?

a Yes

b No

c Prefer not to say

1. Is English your first language?

a Yes

b No

c Prefer not to say

1. Do you have any of the following?

a Deafness or severe hearing impairment

b Blindness or severe vision impairment

c Chronic pain lasting at least 3 months

d A mental health condition

e A learning disability

f Another long term condition

g A physical disability

h None of the above

I Prefer not to say

1. What is your highest level of education?

a None

b Primary school

c GCSE / O-levels / Scottish Standard Grades / NVQ / BTEC

d A-levels / Scottish Highers and Advanced Highers / IB

e Undergraduate degree (e.g. Bachelor’s)

f Postgraduate degree (e.g. Master’s/PGCE)

g Doctorate

h Prefer not to say

1. Employment status: Are you typically…?

If you are currently on maternity leave, please answer this question with your employment status before you went on maternity leave

a Employed for wages

b Self-employed

c Out of work and looking for work

d Out of work and not looking for work

e A student

f A homemaker

g Retired

h Unable to work

i None of the above

j Prefer not to say

# Final page

Thank you for taking the time to complete this survey. Your responses will help the NHS improve maternity services for women in the future.

We are also inviting women to share their views and experiences of maternity care with us in more detail. We will select a range of women from different backgrounds to ensure that we include a wide range of views. We will invite these women to take part in an online interview with a researcher at the University of Manchester.

If you are happy for us to contact you with more details, please enter your name and email address below. Please only provide your details if you wish to be contacted.

Only the research team will have access to your contact details for the purpose of contacting you with more information about this study. We will not share them with any third parties. We will only store your details for the duration of the study, after which they will be destroyed.

I am happy to be contacted if I am selected for interview:

Name:

Email address:


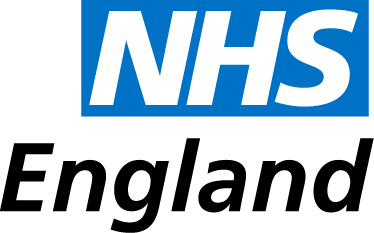

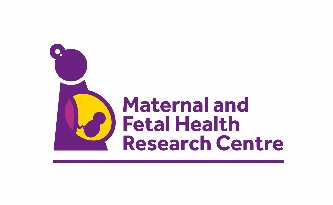

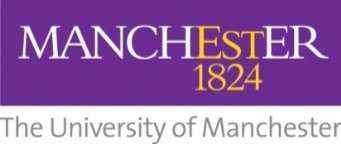


**Saving Babies Lives Care Bundle 2 Survey**

**What is this survey about?**

You are invited to take part in a survey being carried out by the University of Manchester on behalf of NHS England. You are being invited because you are currently a healthcare professional working in NHS maternity services. The survey asks about your views and experiences of the implementation of the **Saving Babies Lives care Bundle Version 2,** and the impact on your own practice and the services provided by your maternity unit.

Taking part is **voluntary** and **anonymous.** Choosing to take part will not disadvantage you in anyway.

**What is my information used for?**

**Your views are very important to us**. Your responses will help the NHS improve future iterations of the Care Bundle and consequently maternity care across the country. We may share a summary of responses with hospital Trusts to understand how they can improve local maternity services in future. It will not be possible to identify you from the summary responses and your responses will not be shared with Trusts other than your own.

**Am I eligible to take part?**

All healthcare professionals who deliver the care bundle interventions as part of maternity care are eligible. This includes, midwives, healthcare practitioners, maternity support workers, sonographers, junior doctors, consultant obstetricians.

**What do I need to do?**

You will be asked to complete an online survey. It should take approximately 10-20 minutes to complete. Firstly, you will be asked some questions about your role. The survey will then ask you about your views and experiences of the care bundle as a whole and its implementation in practice. Finally, it will ask you questions about yourself, such as age and ethnicity.

Once you have completed the survey, you will be given the option to provide your contact details if you are interested in taking part in a short follow-up online interview and would like the researchers to contact you. Contact details will be kept strictly confidential and stored separately to your survey responses. You do not have to provide your contact details if you do not want to.

**Can I withdraw from the study?**

Because your responses are anonymous, once you submit your responses, you will not be able to withdraw them. Thus, by completing the survey you understand your answers cannot be withdrawn and will be used in this study.

If you provide your contact details, we will review your surveys responses in order to select certain groups of healthcare professionals to interview. This is so we can ensure we have heard the views of a wide range of service providers from different roles and levels of experience. This does not affect your survey responses which will be analysed anonymously.

**What will happen to the information I supply?**

Your responses will be analysed by the research team at the University of Manchester. The results will be published on the NHS England website at the end of the study. Once the study has ended we will keep your answers for 5 years in line with the University of Manchester’s recommendations. The information may be looked at by responsible individuals at the University of Manchester and regulatory authorities. Any contact details you supply will only be kept until the end of the study and then destroyed. We will not pass them on to any third parties. Anonymous survey responses will be permanently stored in the University’s data repository.

**Can I get more information about the study?**

If you have any queries about the study or have any questions please contact the research team at the University of Manchester or your healthcare professional.

Kate Widdows: [Kate.Widdows@manchester.ac.uk](mailto:Kate.Widdows@manchester.ac.uk)

Debbie Smith: [Debbie.Smith-2@manchester.ac.uk](mailto:Debbie.Smith-2@manchester.ac.uk)

**You do not have to complete the questionnaire if you do not want to.**

# Section A – About your professional role

Thank you for agreeing to take part in this survey. First, we would like to ask you some questions about your professional role.

1. In which Trust do you work?

Drop down list of Trusts

1. What is your role?

a Midwife

b Doctor

c Sonographer

d Healthcare practitioner

e Maternity support worker

d Other

If d, please specify

1. In which setting(s) do you work

*Tick all that apply*

a Antenatal clinic

b Antenatal ward

c Maternity assessment or triage

d Early pregnancy unit

e Labour ward or birth centre

f Postnatal ward

g Neonatal unit

h Community

i Ultrasound department

j Fetal medicine department

k Practice education

l Other, please specify:

# Section B – Adoption of the care bundle

We would now like to know about the adoption of the care bundle in your maternity unit.

1. Which elements of the care bundle is your organisation implementing?

*Tick all that apply*

a Element 1 - Reducing smoking pregnancy

b Element 2 - Risk assessment, prevention and surveillance of pregnancies at risk of fetal growth restriction (FGR)

c Element 3 - Raising awareness of reduced fetal movement (RFM)

d Element 4 - Effective fetal monitoring during labour

e Element 5 - Reducing preterm birth

f All of the above

g Don't know

1. How long has your organisation been implementing Version 2 of the Saving Babies Lives Care Bundle?

a Less than 1 year

b 1-2 years

c 3-4 years

d More than 4 years

e I don’t know

1. Overall, how confident are you that you understand your role in implementing the Saving Babies' Lives Care Bundle?

a Very confident

b Quite confident

c Slightly confident

d No feelings

e Slightly unconfident

f Quite unconfident

g Very unconfident

1. Overall, how would you describe your experience of implementing the Saving Babies' Lives Care Bundle?

a Very positive

b Quite positive

c Slightly positive

d No feelings

e Slightly negative

f Quite negative

g Very negative

# Section C - Your views of the care bundle as a whole

We would now like to know your views and experience of the Saving Babies Lives Care Bundle Version 2 **as a whole**. Please read the statements below and indicate how much you disagree or agree where relevant.

To what extent do the following statements reflect your **view** of the care bundle as a whole?

*a. Strongly disagree b. Disagree c. Neither agree nor disagree d. Agree e. Strongly agree*

1. The interventions are clear and understandable to me
2. I have not studied the care bundle interventions in detail
3. I lack knowledge to apply the interventions
4. I have not had enough training in the skills needed to implement the interventions
5. The care bundle is too complex to implement fully
6. The care bundle is evidence-based

To what extent do the following statements reflect your **feelings** towards the care bundle as a whole?

*a. Strongly disagree b. Disagree c. Neither agree nor disagree d. Agree e. Strongly agree*

1. The care bundle is effective at meeting its objectives
2. I agree with the aims of the care bundle
3. I disagree with the recommendations in the care bundle
4. I support the implementation of the care bundle in my organisation
5. Has your confidence in the care bundle to deliver its aims changed over time?

a Grown over time

b Reduced over time

c Stayed the same

d Don't know

To what extent do you the following statements reflect your **experience** of implementing the care bundle?

*a. Strongly disagree b. Disagree c. Neither agree nor disagree d. Agree e. Strongly agree*

1. The intervention(s) take too much time
2. There is not enough staff to enable me to do my job properly
3. My colleagues often disregard the interventions in the care bundle
4. I have difficulty in changing my old routines
5. The care bundle is not much different to my normal routine practice
6. The care bundle supports my clinical reasoning and decision making
7. The care bundle allows me to take into account the patient's wishes

How has the **implementation** of the care bundle impacted on your daily practice?

*a. Strongly disagree b. Disagree c. Neither agree nor disagree d. Agree e. Strongly agree*

1. My workload has increased
2. I work longer hours
3. I have less time with each patient
4. I feel I provide a better level of care
5. My relationship with patients has improved

To what extent do you agree/disagree with the following statements?

*a. Strongly disagree b. Disagree c. Neither agree nor disagree d. Agree e. Strongly agree*

1. Leadership is driving the implementation of the care bundle in my organisation
2. My colleagues support the adoption of the care bundle within my organisation
3. My managers support the adoption of the care bundle within my organisation
4. Information about roll out of version 2 was communicated well within my organisation
5. The care bundle is now embedded in routine practice in my organisation

In your organisation, over the last 5 years (or since you've been in post) do you perceive that

*a. Greatly increased b. Slightly increased c. Not changed d. Slightly decreased e. Greatly decreased*

1. The number of inductions has
2. The number of caesareans has
3. The number of preterm births has
4. The number of stillbirths has
5. The number of neonatal deaths has
6. The number of babies admitted to neonatal care has
7. The number of babies dying following admission to neonatal care has
8. The number of incidents has

# Section D - Element 1: Reducing smoking in pregnancy

Please tell us about your experience of implementing Element 1 of the care bundle (e.g. carbon monoxide testing, referral to smoking cessation services)

1. Are you involved in implementing interventions in Element 1?

a Yes

b No

c Don't know

If b, skip to question 55

1. Overall, how competent do you feel to implement this element?

a Very competent

b Quite competent

c Slightly competent

d No feelings

e Slightly incompetent

f Quite incompetent

g Very incompetent

1. Overall, how would you describe your experience of implementing this element?

a Very positive

b Quite positive

c Slightly positive

d No feelings

e Slightly negative

f Quite negative

g Very negative

1. Do you offer women a carbon monoxide (CO) breath test during antenatal appointment?

Tick all that apply

a Yes, at booking appointment

b Yes, at 36 weeks appointment or later

c No, we don't use CO monitors

d No, I assess a woman's smoking status verbally

e No, a woman's smoking status is not assessed

To what extent do you agree/disagree with the following statements?

*a. Strongly disagree b. Disagree c. Neither agree nor disagree d. Agree e. Strongly agree*

1. I have enough time during antenatal appointments to carry out CO testing
2. I am adequately trained in CO testing
3. There are enough CO monitors in my work area to ensure all women are tested
4. Effective pathways are in place in my organisation to refer women to a stop smoking specialist
5. I have enough time to record CO measurements in patients notes (handheld or electronic)
6. Women are receptive to CO testing
7. If you don't record a woman's CO measurement in your hospital or maternity information system (MIS), why not?

*Tick all that apply*

a Lack of time

b Difficulties in accessing a computer

c It's not in our guidelines to record CO measurements in our MIS

d Our MIS doesn't enable this

e I record CO measurements in women’s hand-held notes

f I often forget

g Other

# Section E - Element 2: Risk assessment, prevention and surveillance of pregnancies at risk of fetal growth restriction

Please tell us about your experience of implementing the interventions in Element 2 of the care bundle (e.g. risk assessment and triage of women at increased risk of FGR, management of the SGA and growth restricted fetus).

1. Are you involved in implementing interventions in Element 2

a Yes

b No

If b, skip to question 74

1. Overall, how competent do you feel to implement this element?

a Very competent

b Quite competent

c Slightly competent

d No feelings

e Slightly incompetent

f Quite incompetent

g Very incompetent

1. Overall, how would you describe your experience of implementing this element?

a Very positive

b Quite positive

c Slightly positive

d No feelings

e Slightly negative

f Quite negative

g Very negative

1. Are you involved in assessing a woman's risk of fetal growth restriction (FGR) during antenatal care?

a Yes

b No

If a, display questions 59 and 60

If b, skip to question 61

1. Do you assess women at booking to determine if aspirin should be prescribed?

a Yes, I use the care bundle algorithm (Appendix C)

b Yes, I use an alternative pathway

c No

1. Do you use a risk assessment pathway for triaging women at risk of FGR into an appropriate clinical pathway for surveillance of FGR?

*Tick all that apply*

a Yes, my organisation uses the care bundle algorithm (Appendix D)

b Yes, my organisation has its own algorithm

c Yes, my organisation uses the Growth Assessment Protocol (GROW)

d Yes, other

e No

f Don't know

1. Do you assess fetal growth using symphysis fundal height?

a Yes

b No

If b, skip to question 66

To what extent do the following statements reflect your training and competency in measuring and plotting symphysis fundal height on antenatal growth charts (population or customised)

*a. Strongly disagree b. Disagree c. Neither agree nor disagree d. Agree e. Strongly agree*

1. I am adequately trained in measuring symphysis fundal height
2. I feel competent in plotting symphysis fundal height
3. I feel competent in interpreting antenatal growth charts
4. I feel competent in referring women when indicated
5. Do you carry out ultrasound scans to assess fetal growth (e.g. fetal biometry, EFW or uterine artery Doppler)

a Yes

b No

If b, skip to question 72

How do you perceive current capacity for ultrasound scans in your organisation?

*a. Strongly disagree b. Disagree c. Neither agree nor disagree d. Agree e. Strongly agree*

1. We have enough ultrasonographers to scan all women referred
2. The demand for ultrasound scans has increased
3. Women are being referred for scans unnecessarily
4. The demand for uterine artery Doppler scans has increased
5. We don't have enough staff trained to carry out uterine artery Doppler scans
6. If you don't record antenatal detection rates of SGA/FGR in your maternity information system (MIS), why not?

a Lack of time

b Difficulties in accessing a computer

c It's not in our guidelines to record this in our MIS

d Our MIS doesn't enable this

e We use another system (e.g. GROW)

f Other

1. In your opinion, how do you feel the care bundle has influenced antenatal detection rates of SGA/FGR babies in your organisation?

a Greatly improving

b Slightly improving

c Not changed

d Slightly worsening

e Greatly worsening

f I don't know the SGA/FGR detection rates in my organisation

# Section F - Element 3: Raising awareness of reduced fetal movements

Please tell us about your experience of implementing the interventions in Element 3 of the care bundle (e.g. information leaflet on RFM, checklist for managing RFM)

1. Are you involved in delivering interventions in Element 3?

a Yes

b No

If b, skip to question 81

1. Overall, how competent do you feel to implement this element?

a Very competent

b Quite competent

c Slightly competent

d No feelings

e Slightly incompetent

f Quite incompetent

g Very incompetent

1. Overall, how would you describe your experience of implementing this element?

a Very positive

b Quite positive

c Slightly positive

d No feelings

e Slightly negative

f Quite negative

g Very negative

1. Do you give women an advice leaflet on reduced fetal movements (RFM) by 28 weeks of pregnancy?

a Yes, the Tommy's advice leaflet on RFM

b Yes, my organisation has its own advice leaflet

c No

1. How often do you discuss monitoring baby’s movements and/or RFM with women during their antenatal care?

a At all subsequent appointments

b At some appointments

c I don’t discuss this further with women

1. Do you use a checklist to manage women who report RFM in your organisation?

a Yes, I use the checklist in the care bundle

b Yes, my organisation has its own checklist

c No

1. In your opinion, what is the biggest barrier for women attending antenatal triage for RFM?

a Lack of time

b Feelings of being a burden

c Lack of knowledge regarding risks

d Transport issues

f Childcare issues

g Cultural values (e.g. women listening to advice from family/friends rather than their midwife/doctor)

# Section G - Element 4: Effective fetal monitoring during labour

Please tell us about your experience of implementing the interventions in Element 4 of the care bundle (e.g. annual training and competency assessment on cardiotocograph (CTG) interpretation and use of auscultation)

1. Are you involved in delivering interventions in Element 4?

a Yes

b No

If b, skip to question 93

1. Overall, how competent do you feel to implement this element?

a Very competent

b Quite competent

c Slightly competent

d No feelings

e Slightly incompetent

f Quite incompetent

g Very incompetent

1. Overall, how would you describe your experience of implementing this element?

a Very positive

b Quite positive

c Slightly positive

d No feelings

e Slightly negative

f Quite negative

g Very negative

1. Do you care for women in labour?

a Yes

b No

1. Have you received training in intermittent auscultation (IA)?

a Yes, annual training

b Yes, competency assessment

c Both

d I've not had training

e Don't remember

In your experience….

*a. Strongly disagree b. Disagree c. Neither agree nor disagree d. Agree e. Strongly agree*

1. I feel competent in the use of intermittent auscultation
2. I would like more training in intermittent auscultation
3. I feel confident knowing when to transition from IA to CTG

1. Have you received training in CTG interpretation in the last 12 months?

*Tick all that apply*

a Yes, annual training

b Yes, competency assessment

c I've not had either

d Don’t remember

When carrying out fetal monitoring in labour….

*a. Strongly disagree b. Disagree c. Neither agree nor disagree d. Agree e. Strongly agree*

1. I feel competent in interpreting CTG
2. I feel confident in recognising the transition from normal to abnormal CTG
3. I know which member of staff to escalate concerns to when the CTG is abnormal

# Section H - Element 5: Reducing preterm birth

Please tell us about your experience of implementing the interventions in Element 5 of the care bundle (e.g. assessing women’s risk of preterm birth and stratify to low, intermediate and high risk pathways, prescribing corticosteroids and magnesium sulphate to women at imminent risk of preterm birth).

1. Are you involved in delivering interventions in Element 5

a Yes

b No

If b, skip to question 102

1. Overall, how competent do you feel to implement this element?

a Very competent

b Quite competent

c Slightly competent

d No feelings

e Slightly incompetent

f Quite incompetent

g Very incompetent

1. Overall, how would you describe your experience of implementing this element?

a Very positive

b Quite positive

c Slightly positive

d No feelings

e Slightly negative

f Quite negative

g Very negative

When assessing a woman's risk of preterm birth….

*a. Strongly disagree b. Disagree c. Neither agree nor disagree d. Agree e. Strongly agree*

1. I am confident stratifying women to low, intermediate and high risk pathways for preterm birth
2. I am confident when to refer women to tertiary services, including preterm birth prevention clinics
3. I am competent in offering appropriate interventions to women who are identified as high-risk (e.g. cervical cerclage, pessary and progesterone)
4. I am competent knowing when to prescribe corticosteroids
5. I am competent knowing when to prescribe magnesium sulphate
6. What guidelines do you use to assess a woman's risk of preterm birth?

*Tick all that apply*

a Risk assessment tool from the care bundle (Appendix F)

b NICE guidance

c UK Preterm Clinical Network Guidance

d Local guidelines

e Other

f None

# Section I - About you

1. What is your current post?

a Consultant Obstetrician

b Consultant in Fetomaternal Medicine

c Speciality Trainee 6-7

d Speciality Trainee 3-5

e Speciality Trainee 1-2

f Subspecialty Trainee in Fetomaternal Medicine

g Head of Midwifery

h Midwife Band 8 (not regularly working in the community)

j Midwife Band 8 (regularly working in the community)

k Midwife Band 7 (not regularly working in the community)

l Midwife Band 7 (regularly working in the community)

m Midwife Band 6 (not regularly working in the community)

n Midwife Band 6 (regularly working in the community)

o Midwife Band 5 (not regularly working in the community)

p Midwife Band 5 (regularly working in the community)

q Ultrasonographer Band 8

r Ultrasonographer Band 7

s Ultrasonographer Band 6

t Other (Please specify)

1. How many years have you worked for this organisation?

a Less than 1 year

b 1-2 years

c 3-5 years

d 6-10 years

e 11-15 years

f More than 15 years

1. Were you recruited from outside the UK (international recruitment)?

a Yes

b No

c Prefer not to say

1. What is your age group?

a 16-20

b 21-30

c 31-40

d 41-50

e 51-65

f 66+

1. What best describes your ethnic group?

White

a British

b Irish

c Other

Mixed

d White and Black Caribbean

e White and Black African

f White and Asian

g Other

Asian or Asian British

h Indian

i Pakistani

j Bangladeshi

k Other

Black or Black British

l Caribbean

m African

n Other

Other ethnic groups

o Chinese

p Other

q Prefer not to say

1. Do you have any of the following?

a Deafness or severe hearing impairment

b Blindness of severe vision impairment

c Chronic pain lasting at least 3 months

d A mental health condition

e A learning disability

f Another long term condition

g A physical disability

h None of the above

I Prefer not to say

**End of survey**

# Final page

Thank you for taking the time to complete this survey. Your responses will help the NHS improve maternity services for women in the future.

We are also inviting healthcare professionals to share their views and experiences of maternity care with us in more detail. We will select a range of healthcare professionals from different roles and experience levels to ensure that we include a wide range of views. We will invite these healthcare professionals to take part in an online interview with a researcher at the University of Manchester.

If you are happy for us to contact you with more details, please enter your name and email address below. Please only provide your details if you wish to be contacted.

Only the research team will have access to your contact details for the purpose of contacting you with more information about this study. We will not share them with any third parties. We will only store your details for the duration of the study, after which they will be destroyed.

I am happy to be contacted if I am selected for interview:

Name:

Email address:
